# Persister cells in human fungal pathogens

**DOI:** 10.1371/journal.ppat.1013483

**Published:** 2025-10-23

**Authors:** Yuyan Xie, Weixin Ke, Koon Ho Wong, Linqi Wang

**Affiliations:** 1 State Key Laboratory of Microbial Diversity and Innovative Utilization, Institute of Microbiology, Chinese Academy of Sciences, Beijing, China; 2 University of Chinese Academy of Sciences, Beijing, China; 3 Faculty of Health Sciences, University of Macau, Macau SAR, China; 4 Institute of Translational Medicine, Faculty of Health Sciences, University of Macau, Macau SAR, China; 5 MoE Frontiers Science Center for Precision Oncology, University of Macau, Macau SAR, China; University of Maryland, Baltimore, UNITED STATES OF AMERICA

## Abstract

Microbicidal persistence refers to the phenomenon whereby a subpopulation of microbial cells enters a dormant state to evade drug killing. This phenomenon is associated with antibiotic treatment failure in bacterial infections, and thus, the characteristics and mechanisms of bacterial persistence have been extensively studied. Despite significant evolutionary divergence, microbicidal persistence has also been observed in fungi. Notably, recent studies have demonstrated that fungal persistence can occur within the host and significantly impair the efficacy of fungicidal drugs. Given the extremely limited range of first-line fungicidal agents currently in use, improving our understanding of the shared and unique mechanisms of antifungal persistence in various fungal pathogens is of great clinical importance. This review summarizes recent advances in the study of antifungal persistence, covering conceptual definitions, measurement methods, and molecular mechanisms. It also discusses future research directions in this field.

## Introduction

The incidence and mortality of invasive fungal diseases (IFDs) have increased significantly in recent decades, driven largely by evolving human lifestyles and shifting disease patterns. This trend is particularly pronounced among immunocompromised populations, which have expanded dramatically in size [1]. Recent estimates indicate that IFDs are responsible for approximately 3.75 million annual deaths worldwide, causing a major global public health challenge [2].

Despite this substantial disease burden, the antifungal therapeutic arsenal remains alarmingly limited. Currently, only three major classes of antifungal agents are available for monotherapy against IFDs: (1) polyenes (e.g., amphotericin B [AmB]), which target fungal cell membranes by binding to ergosterol; (2) azoles (e.g., fluconazole, itraconazole, voriconazole), which inhibit ergosterol biosynthesis; and (3) echinocandins (e.g., caspofungin, micafungin), which disrupt fungal cell wall polysaccharide synthesis [3]. Among these, AmB functions as a broad-spectrum fungicidal agent capable of killing fungal cells, whereas most other antifungals exhibit fungistatic activity, merely inhibiting fungal growth without achieving cell death [4].

**Fig 1:**
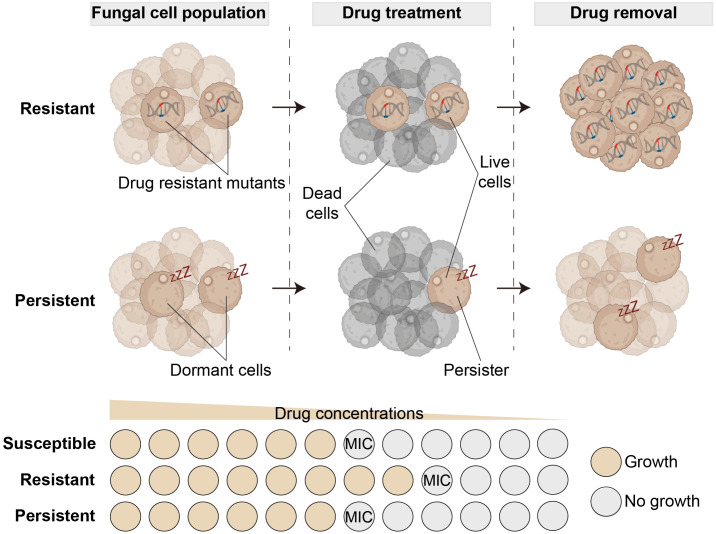
The conceptual difference between antifungal resistance and antifungal persistence. Antifungal resistance arises from genetic mutations in drug resistance-associated genes, leading to a measurable increase in the minimum inhibitory concentration (MIC) value. In contrast, antifungal persistence typically results from cellular dormancy that enables fungal cells to survive lethal drug concentrations without a change in MIC value. The figure was generated in BioRender.

The widespread clinical use of antifungals in the clinical and agricultural settings has led to the emergence of drug-resistant variants in different fungal pathogens [5]. This resistance is mediated through genetically heritable mechanisms that allow fungi to survive drug exposure, typically reflected by elevated minimum inhibitory concentration (MIC) values (Fig 1) [3]. Notably, treatment failures in IFDs remain frequent, often occurring even in cases caused by non-drug-resistant or even drug-susceptible isolates [6]. While host determinants (e.g., immune status) have traditionally been implicated in this “drug susceptible–treatment failure” paradox, emerging research highlights the critical role of alternative fungal survival strategies beyond classical resistance mechanisms [6].

Fungal pathogens can also evade fungicidal drugs (e.g., AmB) through survival mechanisms such as tolerance and persistence to fungicidal drugs, in both *in vitro* and *in vivo* settings [3,7–9]. Animal studies suggest that these underappreciated survival strategies may contribute to treatment failures, emphasizing the need to expand research beyond conventional resistance paradigms. In this regard, fungicidal persistence has been observed across phylogenetically diverse fungal pathogens, suggesting this phenomenon may be widespread among fungal diseases [10–15]. In this review, we systematically integrate recent progress in understanding fungicidal persistence, encompassing its conceptual framework, methodologies for *in vitro* and *in vivo* evaluation, and underlying molecular mechanisms. We also discuss key challenges and unresolved questions in antifungal persistence research, providing a comprehensive framework to guide future studies and therapeutic development. Due to space constraints, we are unable to cite all the relevant studies in this field, and we apologize to the authors whose work could not be included.

## Definition and evaluation of antifungal persistence

Unlike drug resistance, tolerance and persistence reflect the ability of genetically susceptible bacterial cells to survive high doses of bactericidal antibiotics without altering the MIC [3]. While the terms “persistence” and “tolerance” are often conflated in the literature, Balaban *and colleagues’* consensus statement provides a conceptual distinction: tolerance represents a population-wide phenotype to survive antibiotic treatment, whereas persistence describes a small, metabolically dormant subpopulation that survives exposure to high concentrations of bactericidal antibiotics [16]. This population heterogeneity manifests as biphasic killing kinetics, where persisters and other cells within the same population exhibit differential killing rates under antibiotic challenge [16]. Critically, this population heterogeneity should be distinguished from another phenomenon called heteroresistance, which describes the coexistence of a minor subpopulation of resistant cells displaying a higher MIC and a majority of susceptible cells [17]. Although fungi are evolutionarily distant from bacteria, the concept of drug persistence has been extended to fungi based on similar observed phenomena (Fig 1 and S1 Table) [9].

A classical method for evaluating antifungal persistence is the time-kill curve assay, which typically employs the minimum duration for killing 99.99% of fungal cells (MDK99.99) to quantify the level of drug persistence [16]. Alternatively, persistence can also be determined by evaluating the survival rate through colony-forming unit counting or live/dead staining, which is the most commonly used methods for assessing antifungal persistence [12,14,15,18]. The aforementioned detection methods are typically applied to homogeneous dormant cell populations *in vitro*, often obtained from stationary-phase cultures. However, their application becomes limited when studying microbial persistence *in vivo* due to the inherent physiological heterogeneity of infecting populations, which complicates the identification and isolation of dormant subpopulations. To overcome this challenge, dormancy-labeling and cell-tracking strategies offer a promising solution for detecting and isolating dormant fungal cells in complex *in vivo* environments. For instance, Ke *and colleagues* identified Sps1, a dormancy-specific protein marker in *Cryptococcus neoformans*, and engineered a reporter strain expressing a fluorescently tagged Sps1 fusion protein [14]. This system enables direct detection and isolation of dormant fungal subpopulations *in vivo*, facilitating the assessment of their persistence activity against AmB treatment [14].

Despite recent advances in assessing fungal persistence both *in vitro* and *in vivo*, a fundamental challenge remains in comprehensively understanding the determinants of fungal persistence. This limitation stems partially from the lack of high-throughput strategies capable of systematically evaluating antifungal persistence levels across diverse clinical isolates or genome-edited strain libraries. In light of the growing recognition of the clinical relevance of antifungal persistence, developing robust, high-throughput screening platforms would greatly improve our ability to use pan-omics or functional genomics approaches in mechanistic studies and clinical applications of antifungal persistence research.

## Discovery and evidence of antifungal persistence

The phenomenon of drug persistence was initially described in bacteria in the 1940s [9]. Over the past decades, bacterial persistence has been extensively investigated due to its close association with recurrent and persistent bacterial infections [9]. Although recurrent and persistent infections are also frequently observed during antifungal therapy, the recognition of antifungal persistence occurred significantly later than in bacteria. This delay primarily stems from the relatively late clinical attention given to fungal infections. In 2006, LaFleur *and colleagues* reported persister cells in biofilms of *C. albicans* [15]. Since then, the phenomenon of antifungal persistence has been observed in various fungal pathogens, including *Candida tropicalis*, *C. krusei*, *C. parapsilosis*, and *Aspergillus fumigatus* [11,13,19].

In recent years, the identification of *ex vivo* or *in vivo* evidence of antifungal persistence has further highlighted its clinical importance. For instance, Arastehfar *and colleagues* demonstrated that macrophage-induced oxidative stress promotes the formation of persisters that are highly tolerant to multiple fungicides [10]. Furthermore, our recent research has provided *in vivo* evidence of the formation of a highly AmB-tolerant persister population in pulmonary cryptococcosis caused by *C. neoformans* [14].

These studies provide a plausible explanation for the clinically observed “drug susceptible-treatment failure” paradox. However, the clinical relevance of antifungal persistence in most fungal diseases remains unclear due to the lack of clinical cohort studies. Despite this, a few retrospective studies have suggested the clinical relevance of antifungal persistence. An important example is the study on oral candidiasis conducted by LaFleur *and colleagues* [18]. They collected clinical isolates of *C. albicans* from high-risk patients susceptible to oral candidiasis, demonstrating that strains isolated from patients with long-term carriage had high levels of persisters [18]. Rasilla *and colleagues* have also revealed the relevance of antifungal persistence to the treatment failure of *A. fumigatus* infections. They described two hematological patients infected by classical “drug-susceptible” strains who failed antifungal therapy despite adequate drug exposure. Microbiological analysis of the recovered infective isolates showed that the patients were infected with multiple strains, the majority of which exhibited antifungal persistence to voriconazole and/or itraconazole. The observations strongly implicate antifungal persistence in clinical treatment outcomes. To determine whether antifungal persistence contributes broadly to therapeutic failure across diverse fungal infections, we propose a three-stage clinical study comprising: (1) collection of clinical fungal isolates with concomitant documentation of patient treatment response data; (2) development of a robust high-throughput screening platform for quantitative assessment of persister levels across diverse clinical isolates; and (3) application of multivariate statistical analysis to evaluate correlations between persister levels and clinical outcomes.

## Mechanisms of antifungal persistence

Compared to the extensive research on drug resistance mechanisms in fungi, less is known about persistence mechanisms. Antifungal persistence is most commonly observed in biofilm microenvironments. Notably, a large proportion of cells in biofilms exhibit non-proliferative characteristics, supporting the potential role of dormant or dormancy-like states in promoting antifungal persistence [8]. Proteomic analyses have revealed significant downregulation of enzymes involved in core energy metabolism pathways in *C. albicans* persister cells, including glycolysis and the tricarboxylic acid (TCA) cycle [20]. This global metabolic suppression typically drives cells into a dormant state, accompanied by a sharp decline in intracellular adenosine triphosphate (ATP) levels. Studies have confirmed that such metabolic reprogramming can promote the formation of AmB-tolerant persisters in various pathogenic fungi [14,20,21].

As a critical driver of antifungal persistence, cellular dormancy can be induced by multiple stressors, with nutrient starvation being one of the most representative triggers. Mechanistic studies in *Saccharomyces cerevisiae* have provided compelling evidence: under nutrient-deprived conditions, the target of rapamycin complex 1 (*TORC1*) signaling pathway activity is markedly suppressed, driving cells into a dormant state and significantly enhancing their persistence against AmB [21].

AmB, owing to its broad-spectrum fungicidal activity, represents the most extensively studied antifungal in persistence research. Its well-characterized mechanisms of action involve dual pathways: direct interaction with ergosterol in fungal membranes and induction of oxidative stress [3]. Studies have revealed that *C. albicans* persister cells downregulate the expression of ergosterol biosynthesis genes, which likely results in fewer AmB target molecules and consequently decreases the drug’s efficacy against persister cells [20]. Concurrently, enhanced cellular antioxidant capacity also contributes to AmB persistence. In *C. neoformans*, the antioxidant ergothioneine (EGT) has been specifically shown to promote the formation of AmB-tolerant persisters during the stationary phase. Importantly, this EGT-mediated AmB persistence mechanism is evolutionarily conserved across diverse pathogenic fungi [14].

Similarly, the formation of miconazole-tolerant *C. albicans* biofilm persisters is also associated with a powerful antioxidant system. For instance, research by Bink *and colleagues* demonstrated that miconazole-tolerant biofilm persister formation was closely associated with the reactive oxygen species-detoxifying activity of superoxide dismutases (Sods). Notably, double deletion of *SOD4* and *SOD5* genes significantly impaired the formation of miconazole-tolerant biofilm persisters [22].

**Fig 2:**
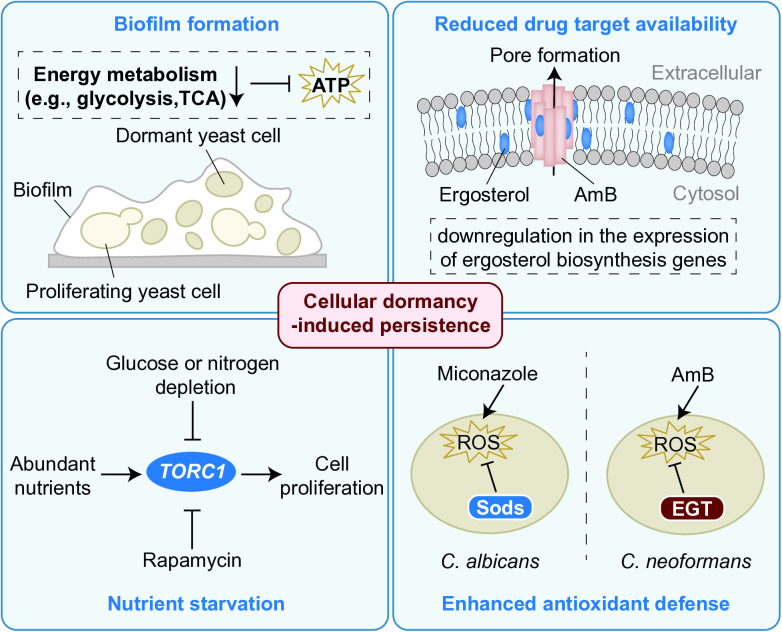
The mechanisms of antifungal persistence. Cellular dormancy is a key driver of antifungal persistence, mainly triggered by biofilm formation and environmental stressors (e.g., nutrient starvation). Persisters evade the action of fungicides primarily through reduced availability of drug targets and enhanced antioxidant defenses.

Additionally, other stress-responsive factors, such as heat shock proteins (HSPs), may participate in antifungal persistence. Studies indicate that HSP family members are markedly upregulated in AmB-tolerant persisters [20]. However, the precise role of HSPs in antifungal persistence remains to be investigated. These findings collectively underscore the complexity and multifaceted nature of the regulatory mechanisms governing antifungal persistence (Fig 2).

## Outlook and perspectives

Over the past two decades, fungal persisters have attracted increasing attention because of their significant clinical implications. Emerging research has revealed that fungal persisters are commonly found in various pathogenic fungi. Despite significant advances in the understanding of fungal persister cells, many questions remain unresolved: what is the correlation between antifungal persistence and clinical outcomes in different fungal diseases? How to establish a consensus assessment standard for evaluating antifungal persistence that is applicable to all species and highly efficient? How do the complex “host–drug–fungus” interactions affect the dynamics of fungal persister formation *in vivo*? Addressing these questions will catalyze new strategies to overcome fungal infections, ultimately improving clinical outcomes.

## Supporting information

S1 Table The definitions, detection methods, and mechanisms of antifungal resistance, tolerance, and persistence. (PDF)
