# Supplementary material for: Persister cells in human fungal pathogens
Source: PLoS Pathog. 2025 Oct 23;21(10):e1013483. doi: 10.1371/journal.ppat.1013483 (PMC12548908; doi:10.1371/journal.ppat.1013483)
Supplement: S1 Table — (PDF) [file ppat.1013483.s001.pdf]

S1 Table. The definitions, detection methods and mechanisms of antifungal resistance, tolerance and persistence.

	Resistance	Tolerance	Persistence
**Definition**	・Proliferation under drug treatment<br>・Genetically heritable<br>・Elevated MIC	・Cell population-wide survival under cidal drug treatment<br>・Genetically non-heritable<br>・Unchanged MIC	・Survival of a small, metabolically dormant cell subpopulation under cidal drug treatment<br>・Genetically non-heritable<br>・Unchanged MIC
**Detection method**	・Broth microdilution assays<br>・Disk diffusion including Epsilometer test (E test)	・Time-kill curve assay (larger MDK99 than susceptible cells)<br>・Survival rate determination	・Time-kill curve assay (larger MDK99.99 than susceptible cells)<br>・Survival rate determination
**Mechanism**	・Mutations in genes including *ERG11*, *FKS1*, *FUR1*, and *FCY2*	・The stress response pathways<br>・Ergosterol availability	・Biofilm formation<br>・Nutrient starvation<br>・Reduced ergosterol synthesis<br>・Enhanced antioxidant defense
